# Characterization of cryo-cooled silicon crystal monochromators *via* measurement of flux *versus* power

**DOI:** 10.1107/S160057752500342X

**Published:** 2025-05-23

**Authors:** Lucia Alianelli, Hossein Khosroabadi, John Sutter, Andrew C. Walters, Pierpaolo Romano, Kalotina Geraki, Francesco Carlá, Jonathan Rawle, Sarah Barnett, Kawal Sawhney

**Affiliations:** a Diamond Light Source Harwell Science and Innovation Campus Didcot OxfordshireOX11 0DE United Kingdom; Brazilian Synchrotron Light Laboratory, Brazil

**Keywords:** silicon crystal monochromator, indirect cryo-cooling, finite element analysis, optics thermal deformation

## Abstract

A study on the thermal load of cryogenically cooled silicon in synchrotron double-crystal monochromators is presented, based on experimental data from four different beamlines at Diamond Light Source. Different amounts of power are deposited on the first monochromator crystal by varying the storage ring current. The resulting crystal deformation causes a decline in the diffraction efficiency when power and power density are above threshold values. The results are compatible with an analytical model of thermo-mechanical deformation. Acceptable monochromator heat load values are determined with this model, to ensure optimal function of the monochromator. This model, previously tested against finite element analyses, is now validated against measured data and it will be used as a tool for initial analysis of monochromator performance on upgraded photon sources.

## Abstract 2

The resilience of recently upgraded double-crystal monochromators under increasing power load at Diamond Light Source is discussed. Experimental evidence and modelling are described.

## 1. Introduction

Following two decades of operation, the Diamond Light Source (DLS) accelerator will be upgraded to a higher energy, lower emittance ring, based on a modified hybrid six-bend achromat (MH6BA) lattice (Chapon *et al.*, 2019 ▸; Ghasem *et al.*, 2024 ▸). User operation will resume in January 2030. The increased capacity of the new Diamond-II machine will allow construction of new beamlines. The horizontal emittance will decrease from the current value of 2.7 nm rad to 160 pm rad. The vertical emittance will remain at current diffraction limit values near 8 pm rad. Lower emittance will translate into increased photon source brightness and increased flux density at the sample on micro- and nano-focus beamlines. An increased electron beam energy, from 3 GeV to 3.5 GeV, will enable higher flux experiments on hard X-ray beamlines. Renewal of undulator sources with higher magnetic field devices, and higher power, is underway. On Diamond-II, nearly all primary optical elements will receive increased power and power density.

Optimal monochromator cooling (Bilderback *et al.*, 2000 ▸) is successfully implemented on modern beamlines (Saveri Silva *et al.* 2016 ▸). In-house design of double-crystal monochromators (DCMs) based on perfect silicon crystals, and medium power levels, has progressed steadily at DLS for 20 years, reaching excellent stability performances. The crystals are indirectly cooled at the sides using liquid nitrogen (LN2) via copper plates and using indium foils as an interface. They are coping well with absorbed power levels ranging from a few tens of Watts up to approximately 200 W. Data in this paper describe the response of some recently upgraded DCMs to increased power, temperature and deformation.

The resilience of DCMs to increased power has been tested previously at DLS (Khosroabadi *et al.*, 2022 ▸). Comprehensive experimental and analytical studies from the European Synchrotron Radiation Facility (Zhang *et al.*, 2013 ▸; Chumakov *et al.*, 2014 ▸) offer details of the deformation process, the temperature variations and effects on the crystal optics. The motivation leading to the tests described in this paper was to provide experimental evidence for the resilience of these DCMs and for the reliability of models described later. The process was the following: (*a*) measure the flux response to increased power and power density on several undulator beamlines at the same time, under the same accelerator conditions, (*b*) determine whether the DCM power load might be the cause for measured flux that is in some cases below theoretical values; (*c*) experimentally demonstrate that the threshold power, below which safe and effective operation of these DCMs is ensured on Diamond-II, can be predicted analytically (Khosroabadi *et al.*, 2024 ▸).

We describe experimental data which confirm the analytical findings. Measurements of diffracted flux versus power were taken on several hard X-ray undulator beamlines to systematically determine whether increasing power will decrease efficiency.

## 2. Definition of power levels leading to critical deformation

The crystal lattice deformation normally present in cryo-cooled silicon is due to different factors: thermo-mechanical response to absorbed power, fabrication imperfections, mounting and clamping (Khosroabadi *et al.*, 2022 ▸; Zhang *et al.*, 2013 ▸; Khosroabadi *et al.*, 2024 ▸). Defining once and for all an acceptable degree of deformation for the crystal lattice is not possible, although, following general and intuitive guidelines, slope r.m.s. values as small as 1 to 2 µrad are often required. Such small values are a fraction of the crystal Darwin width. They are also much smaller than the undulator harmonic angular width, and as a result should not decrease the photon beam brightness. Deformation of Bragg planes below the single micro-radian level is often required for wavefront preservation (Cocco *et al.*, 2022 ▸). The present study aims at exploring whether the power load on current DCMs at DLS causes losses of flux rather than degradation of wavefront quality.

**Figure 1:**
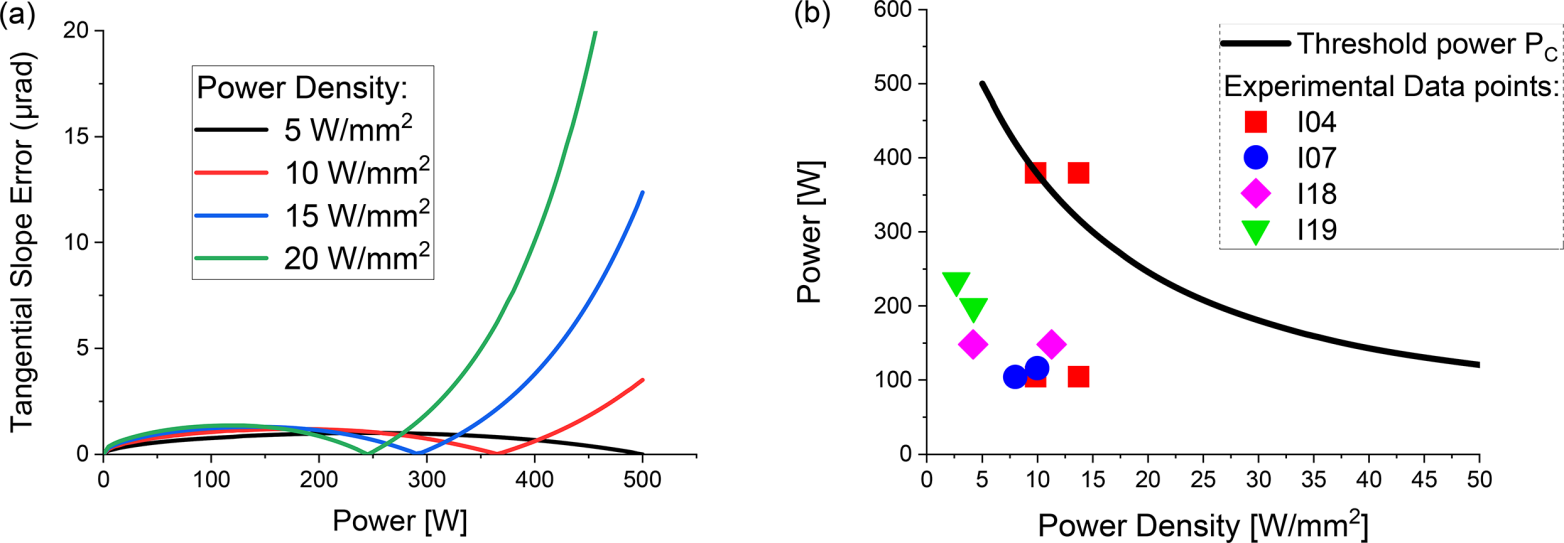
(*a*) Silicon crystal tangential deformation slope using the model of Khosroabadi *et al.* (2024 ▸). Threshold power levels *P*_C_ are defined as values at which deformation starts to increase steeply. (*b*) Experimental power values in Table 1 ▸, plotted along threshold critical power against power spatial density. We have used *K*_Si-Cu_ = 2000 W m^−2^ K^−1^.

Crystal deformation slope error values, caused by absorbed power *P*, are illustrated in Fig. 1 ▸(*a*). Behaviour is affected by the power spatial density on the crystal surface *P*_D_ as well as the cooling design, contact dimensions *etc*. We define the threshold value *P*_C_ above which silicon crystal deformation levels increase steeply and linearly. *P*_C_ is a function of power density *P*_D_ and crystal body temperature *T*. The following relationship is derived from equation (8) of Khosroabadi *et al.* (2024 ▸),
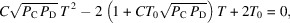
where *C* is a constant, with a small dependence on cooling design. It is mainly determined by Si material properties at cryogenic temperatures and we use the value *C* = 7 × 10^−5^ mm W^−1^ K^−1^ (Khosroabadi *et al.*, 2024 ▸). *T*_0_ ≃ 125 K is the temperature at which silicon thermal expansion is null. Values for *P*_C_ corresponding to DCMs used for this work are plotted in Fig. 1 ▸(*b*). A thermal contact conductance of *K*_Si-Cu_ = 2000 W m^−2^ K^−1^ has been used throughout.

## 3. Experiment

Monochromatic photon flux was measured as a function of the accelerator ring current, in the range 50 mA to 300 mA, on four different beamlines: I04 Variable and Micro-focus Macro-molecular Crystallography (https://www.diamond.ac.uk/Instruments/Mx/I04.html); I07 Surface and Interface Diffraction (Nickilin *et al.*, 2016 ▸); I18 Micro-focus Spectroscopy (Mosselmans *et al.*, 2009 ▸) and I19 Small Molecule Crystal Diffraction (Nowell *et al.*, 2012 ▸). Data were acquired using several types of diagnostics, including calibrated X-ray beam position monitors (XBPMs) and diodes, and an ionization chamber, in positions before and after the focusing optics. Sufficient wait time between current ramps and flux measurements was used to ensure stable temperature crystal conditions. At each ring current value, data were taken in static and identical accelerator beam conditions on all beamlines. *P* and *P*_D_ levels were calculated using *Spectra* (Tanaka, 2021 ▸) and they were determined by insertion device gap, primary slits openings and Bragg angles. A summary is presented in Table 1 ▸ for the highest ring current of 300 mA. The power values are also plotted as symbols in Fig. 1 ▸(*b*). All undulators on these beamlines are 2 m long. Power values differ due to the different technology used in these devices: cryogenic permanent magnet undulators (CPMUs) reaching higher maximum magnetic field than the in-vacuum undulators (IVU). The absorbed power densities also vary with the DCM distance from the undulator, typically between 25 m and 29 m for these beamlines.

A wide range of maximum power values from 104 W to 380 W was used during the experiment. On I04 and I19, the full front-end beam acceptance was used. The I04 data illustrate the DCM response to integrated power levels that are well above the initial design values for this optic. Power on I19 was also higher than typical levels experienced during user operations; however, strong crystal deformation was not expected on I19, due to relatively low Bragg angles and in-vacuum undulator with lower power than the CPMU on I04. Incident power on I18 was higher than in normal user operations due to the large white beam acceptance. I07 power was lowest due to the beamline acceptance being smaller.

## 4. Results and discussion

**Figure 2:**
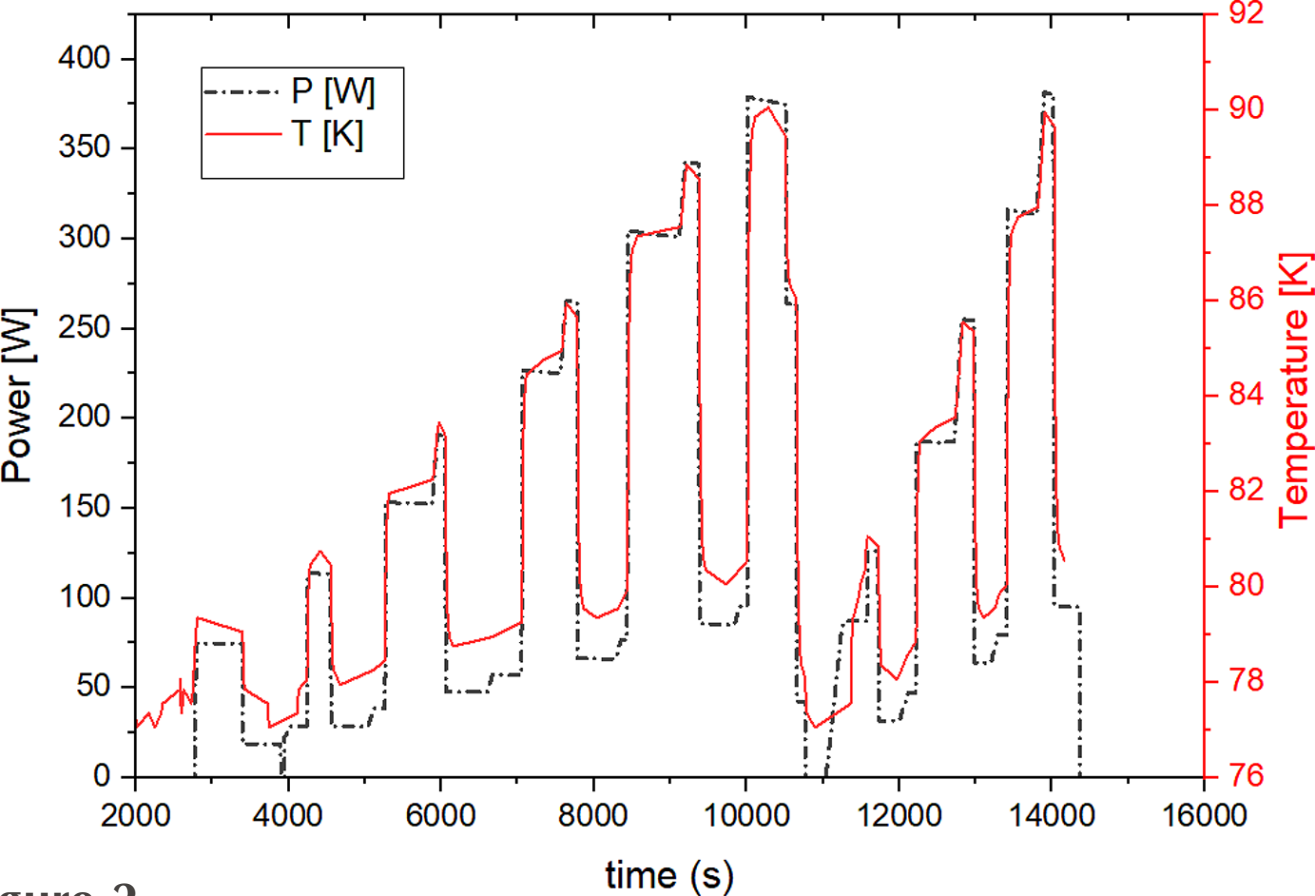
I04 estimated incident power *P* and measured crystal temperature *T*, during the 4 h measurements described in this paper. Power ramps are determined by ring current variations ranging from 50 mA to 300 mA, and variations of the white beam slits sizes, as summarized in Table 1 ▸. The calibrated PT100 temperature sensor is placed in the centre of the non-cooled side and *T* is constantly monitored. Temperature follows power variations, with some lagging due to temporal crystal thermal response. Variations of Bragg angles also determined subtler changes in temperature. Measurements were paused and there was no beam intermittently in the first hour, and then again at 3 h. The crystal temperature was near 77 K, as expected during these pauses.

The crystal monochromators analysed here are thermally very stable, due to high thermal conductivity, efficient contact and cooling. Crystal temperature sensors, placed in the centres of the non-cooled sides, are representative, in very good approximation, of the crystal average temperatures. I04 crystal temperature continuous readings, versus time, are plotted in Fig. 2 ▸. Estimated total power variation is also shown. In addition to ring current variations, primary slits were varied at some points in time. Two different Bragg angles were used during the entire experiment. Changes of power density (with Bragg angle) at constant power are not shown in this plot. Measurements were paused after the initial 3 h and then resumed for the last 50 min. Changes in power follow variations of synchrotron beam current and white beam slit opening. The measured crystal temperature is proportional to power, as expected, with some lagging due to the thermal crystal response. Thermal contact conductance of the copper–silicon interface *K*_Si-Cu_ was retrieved, using the proportionality between *T* and *P*. Using the maximum measured temperature excursion of Δ*T* = 13 K over the baseline temperature 77 K, a contact surface area *A* = 0.014 m^2^ and maximum power *P* = 380 W, we estimate that *K*_Si-Cu_ ≃ 2000 W m^−2^ K^−1^. This indicates good silicon–copper contact and efficient cooling. Also, the measured temperature on I04 is representative of all other DCMs discussed here, due to having the same design.

**Figure 3:**
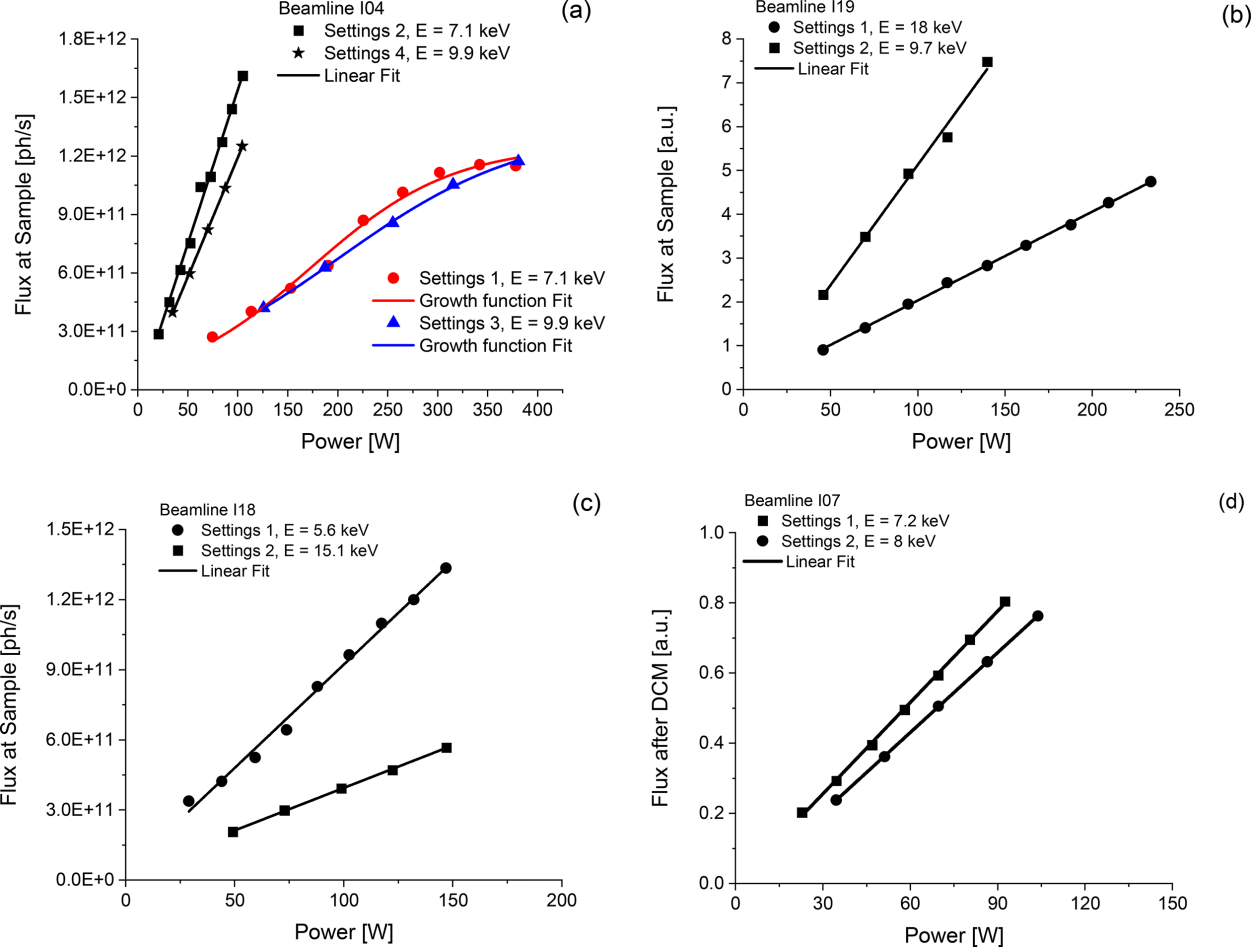
Measured flux as a function of incident power. Data in (*a*), (*b*) and (*c*) are flux values after the focusing optics, *i.e.* at sample position. Data in (*d*) are measured after the DCM, before the focusing optics.

Measured flux versus power is plotted in Fig. 3 ▸. Deviation from linearity is expected at power values crossing above the threshold power. Above such values, the deformation becomes significant. The largest flux losses due to thermal effects are observed on I04 setting 1, *i.e.* a scenario in which the full front-end beam acceptance was used and power density was above 10 W mm^−2^. Crystal deformation is responsible for decreased flux *via*: (1) lower diffraction efficiency due to distortion of the silicon crystal lattice and (2) lower acceptance due to increased incoming beam divergence. I04 uses a beryllium compound refractive lens as micro-focusing optics. At power levels corresponding to the usual beamline acceptance, *i.e.* settings 2 and 4, there is no flux loss. Better linearity is observed in setting 4, where the power density is the lowest. Power values at setting 3, predicted as borderline [Fig. 1 ▸(*b*)], determine a flattening of the flux with current as observed in Fig. 3 ▸(*a*).

The I19 flux at sample using the current undulator is not affected by crystal distortion, as it was already predicted using modelling and finite element analysis (FEA) data. Better linearity is observed in setting 1 (lower power density) than 2. On this beamline, DCM angular scans were performed; the measured rocking curves were nearly independent of ring current and close to expected values. The beam sizes measured with the camera system did not change with ring current. The calibrated measured flux on I19 at one energy point was reported in the beamline paper by Nowell *et al.* (2012 ▸). It appears to be only ∼30% below theoretical values and was not measured again in these tests.

On I18, power values higher than in normal operations were used, leading to no observable flux loss. High power settings were chosen deliberately, to mimic the effect of future increased power on a new photon source. Flux is linear with power; however, linearity is worse for setting 1 corresponding to *P*_D_ = 11.3 W mm^−2^. Calibrated diode measurements are consistent with published flux values (Mosselmans *et al.*, 2009 ▸), which are lower than values calculated using reliable models (Tanaka, 2021 ▸; Rebuffi & Sanchez del Rio, 2016 ▸). Our results show that the low flux issue at the sample is not caused by the DCM.

On I07, monochromatic flux linearity indicates good cryo-cooling efficiency. Absolute flux at sample was also measured: we obtained 4 × 10^12^ photons s^−1^ which is one order of magnitude less than the theoretical value for this beamline. Once again, flux losses here cannot be attributed to the DCM.

By using the concept of threshold power levels illustrated in Figs. 1 ▸(*a*) and 1(*b*), it follows that the only two experimental scenarios with *P* ≥ *P*_C_ are for I04 at full front-end beam acceptance. Only for these two scenarios was a worse DCM performance expected and was indeed measured. We propose further analytical or FEA assessment for Diamond-II scenarios with much higher flux density than used in this work, *i.e.P*_D_ ≥ 20 W mm^−2^. Additional filtering to remove low energy harmonics from the undulator beams has also been recommended. Hard X-ray beamlines in which filtering is not feasible due to the energy range extending to 2 keV (I09, I16, I18 and I23) will require careful consideration of power issues if their undulators are upgraded to devices with higher magnetic fields.

In summary, the resilience of existing DCMs at DLS to varying power levels using FEA and analytical methods was assessed before performing the experiment. Power settings were determined, in terms of white beam acceptance and filtering, for future effective DCM operations on Diamond-II. An analytical model, that can bypass FEA (Khosroabadi *et al.*, 2022 ▸; Khosroabadi *et al.*, 2024 ▸), was used to conclude that the recently upgraded DCMs will cope with the increased power. Such a method can be used to predict crystal deformation in a wide range of settings for many of the Diamond-II DCMs.

## 5. Summary and conclusions

The DCMs tested for this work were replaced in recent years, and do not suffer from wear and tear. Experimental results show that they cope well with higher power. Linear flux response to varying power indicates that, if lower-than-expected flux is measured on beamlines, this is not caused by the monochromator surface deformation due to the heat load. Correct functioning of these optics will permit successful delivery of higher flux and brightness beams on hard X-ray beamlines.

The critical power model previously developed can be used to establish power regimes that will lead to increased deformation and loss of brightness. The model correctly predicted that only two experimental scenarios were in the critical deformation regime. The experimental results do indeed show flux levelling off with rising power for these high-power scenarios only.

## Figures and Tables

**Table 1:** Experimental settings and power values, calculated with *Spectra* at ring current *I* = 300 mA. I04 setting 1 is the only setting with power well above critical threshold, as shown in Fig. 1 ▸(*b*)

Beamline	Undulator type and period (mm)	Setting	Undulator magnetic field, *B* (T)	White beam acceptance (µrad)	*E*_DCM_ (keV)	Power, *P* (W)	Power density, *P*_D_ (W mm^−2^)
I04	CPMU 17.6	1	1.34	132 × 81	7.1	380	13.7
		2	1.34	92 × 29	7.1	105	13.7
		3	1.34	132 × 81	9.9	380	9.8
		4	1.34	92 × 29	9.9	105	9.8
I19	IVU 21	1	0.88	151 × 76	18	234	2.67
		2	0.76	151 × 76	9.7	199	4.24
I18	IVU 27	1	0.96	150 × 64	5.6	148	11.3
		2	0.96	150 × 64	15.12	148	4.2
I07	CPMU 17.7	1	0.86	76 × 64	7.2	116	10
		2	0.77	76 × 64	8	104	8
